# High PD-L1 expression is associated with stage IV disease and poorer overall survival in 186 cases of small cell lung cancers

**DOI:** 10.18632/oncotarget.14935

**Published:** 2017-02-01

**Authors:** Yih-Leong Chang, Ching-Yao Yang, Yen-Lin Huang, Chen-Tu Wu, Pan-Chyr Yang

**Affiliations:** ^1^ Department of Pathology, National Taiwan University Hospital and National Taiwan University College of Medicine, Taipei 10002, Taiwan; ^2^ Department of Internal Medicine, National Taiwan University Hospital and National Taiwan University College of Medicine, Taipei 10002, Taiwan; ^3^ Graduate Institute of Pathology, National Taiwan University College of Medicine, Taipei 10002, Taiwan; ^4^ National Taiwan University, Taipei 10617, Taiwan

**Keywords:** small cell lung cancer, immunotherapy, programmed cell-death ligand 1, stage, overall survival

## Abstract

**Background:**

Small cell lung cancer (SCLC) is an aggressive malignancy with a distinct natural history and dismal prognosis. SCLC is characterized as a recalcitrant neoplasm with limited therapeutic options and platinum-based chemotherapy is the treatment of choice. Programmed cell death-ligand 1(PD-L1)-mediated immune escape may be a suitable target for specific therapy, but its role in SCLC is unclear.

**Materials and methods:**

In total, 186 SCLC cases were investigated. Paraffin-embedded tumor sections were stained with a PD-L1 antibody. PD-L1 overexpression was denoted by moderate-to-strong PD-L1 membrane staining in ≥ 5% of tumor cells. Tumor cells and infiltrating lymphocytes were scored separately.

**Results:**

The overall frequency of PD-L1 overexpression, in tumor cells and tumor infiltrating lymphocytes (TILs) was 78.0% and 54.3%, respectively. High tumor PD-L1 expression was significantly correlated with high TIL PD-L1 expression (P=0.001) and stage IV disease (P=0.048). Multivariate analysis revealed that high tumor PD-L1 expression and stage IV disease were two independent risk factors for poor overall survival.

**Conclusions:**

High PD-L1 expression was observed in SCLCs compared with their expression in conventional NSCLCs. The aggressive behavior of SCLC could be partially related to PD-L1-mediated immune escape. High PD-L1 expression correlated with poor prognosis and may provide a rationale for immunotherapy for high-grade SCLC.

## INTRODUCTION

Small cell lung cancer (SCLC) is an aggressive neuroendocrine cancer that secretes and responds to a wide variety of mitogenic peptide growth factors, SCLC accounts for 10-20% of all lung cancers [1]. It displays a distinct natural history with a high proliferative index and an unusually strong predilection for early metastasis [2]. These tumors are very sensitive to chemotherapy and radiotherapy, but are characterized by the relatively rapid appearance of chemo/radioresistance and relapses are common. The five-year survival is approximately 20-25% for stage I-III disease, in which the tumor is confined to one hemithorax, and 5% for stage IV disease, in which the tumor has metastasized beyond one hemithorax [3]. The development of systemic therapeutics over the past 10 years has been disappointing. Therefore, there is a clear need to find new therapeutic strategies to treat SCLC.

Programmed cell death ligand 1 (PD-L1) couples with programmed cell death 1 (PD-1), a coinhibitory receptor on T-cells, to play an important role in the ability of tumor cells to evade the host immune system [4]. In theory, tumor cells that overexpress PD-L1 have the capacity for “ immune escape”, which can result in aggressive behavior. In fact, it has been reported that PD-L1 overexpression in tumor cells is related to worse disease control and treatment outcomes in many other types of cancer [5, 6]. Recently, clinical trials using monoclonal antibodies targeting the PD-1/PD-L1 axis have shown promising antitumor activity in several malignancies, including lung carcinomas. Preliminary data from these trials suggest that tumor PD-L1 expression may predict response to such treatments [7, 8]. In the context of clinical trials, PD-L1 protein expression on tumor cells, as detected by immunohistochemistry is currently the best predictive biomarker [7, 8, 9].

The goal of this study was to conduct a comprehensive investigation of PD-L1 expression in a large series of patients with SCLC and to correlate the expression with clinicopathologic parameters and clinical outcomes.

## RESULTS

### Patient demographics

One-hundred-sixty-seven (89.8%) of the patients were men, and 19 (10.2%) patients were women (Table 1). One-hundred-sixty (86%) patients were smokers. The mean age at diagnosis was 67.1 years (range, 36-89 years). The distribution of SCLC stage among the patients was as follows: stage I-III disease, 74 patients (39.8%) and stage IV disease, 112 patients (60.2%) (Table 1). One hundred forty-six patients received chemotherapy, radiotherapy, or combination chemoradiotherapy. Most of the patients died of disease. One hundred one patients (54.3%) experienced paraneoplastic syndrome (PNS), which presented as hyponatremia (50.5%), hypokalemia (4.3%), hyperkalemia (3.7%), hyperlipidemia (0.5%) or hypomagnesiumia (0.5%). The serum LDH level was higher than the upper normal limit of normal in 98 patients (52.7%), whereas the high serum CEA levels were noted in 56 patients (30.1%).

**Table 1:** Characteristics of patients, PD-L1 expressions and clinicopathologic parameters

Variable	Total	Tumor PD-L1 expression		P Value	TIL PD-L1 expression		P Value
**Positive**	**Negative**	**Positive**	**Negative**
Patient number	186	145 (78.0%)	41 (22.0%)		101 (54.3%)	85 (45.7%)	
Age (year)							
<60	49	29	20		24	25	
≥60	137	116	21	<0.001	77	60	0.407
Sex							
Male	167	132	35		91	76	
Female	19	13	6	0.379	10	9	1.000
Smoking status*							
Smoker	160	126	34		90	70	
Nonsmoker	13	12	1	0.471	8	5	0.779
Necrosis							
≥20%	53	40	13		27	26	
<20%	133	105	28	0.695	74	59	0.626
Stage							
Stage I-III	74	52	22		39	35	
Stage IV	112	93	19	0.048	62	50	0.765
PNS							
Positive	101	81	20		60	41	
Negative	85	64	21	0.479	41	44	0.141
Serum LDH level							
Normal	88	68	20		43	45	
Abnormal	98	77	21	0.861	58	40	0.185
Serum CEA level							
Normal	130	101	29		67	63	
Abnormal	56	44	12	1.000	34	22	0.266
Tumor PD-L1 expression							
Positive	145				88	57	
Negative	41				13	28	0.001
TIL PD-L1 expression							
Positive	101	88	13				
Negative	85	57	28	0.001			

*No information in some of the patients

Abbreviations: PNS, paraneoplastic syndrome; PD-L1, programmed cell death ligand 1; TIL, tumor-infiltrating lymphocytes.

Interestingly, the tumor location in the patients with SCLC had a higher propensity for the left upper lobe (36.0%), followed by the right upper lobe (23.7%). The distribution of tumors in the left lower lobe, right middle lobe and right lower lobe was the same: 13.4% each.

Given the very limited role of surgery in SCLC, most materials were collected via biopsies, including: bronchoscopic biopsy (125/186, 67.2%), sonography-guided biopsy (30/186, 16.1%) or CT-guided biopsy (22/186, 11.8%). Only a few patients underwent surgery: lobectomy (6/186, 3.2%) or wedge resection (3/186, 1.6%).

**Figure 1:**
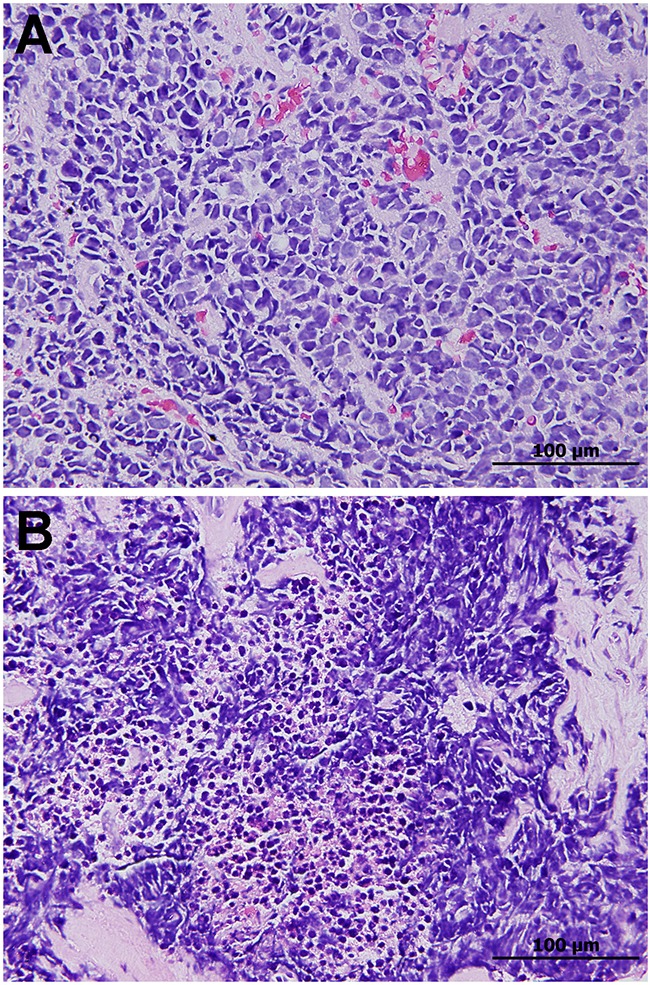
**A**. Small cell lung carcinoma composed of densely packed small tumor cells with scant cytoplasm, finely granular nuclear chromatin, and inconspicuous nucleoli. **B**. Necrosis and brisk apoptotic activity are common.

In the histological analysis, the tumors were characterized by small cells with scant cytoplasm, poorly defined cell borders, finely dispersed granular nuclear chromatin, and absent or inconspicuous nucleoli. The cells were round, oval, or spindle-shaped (Figure 1A). In the 103 tumors of 186 patients (55.4%), necrosis/brisk apoptotic activity was noted (Figure 1B).

### Analysis of PD-L1 expression

**Figure 2:**
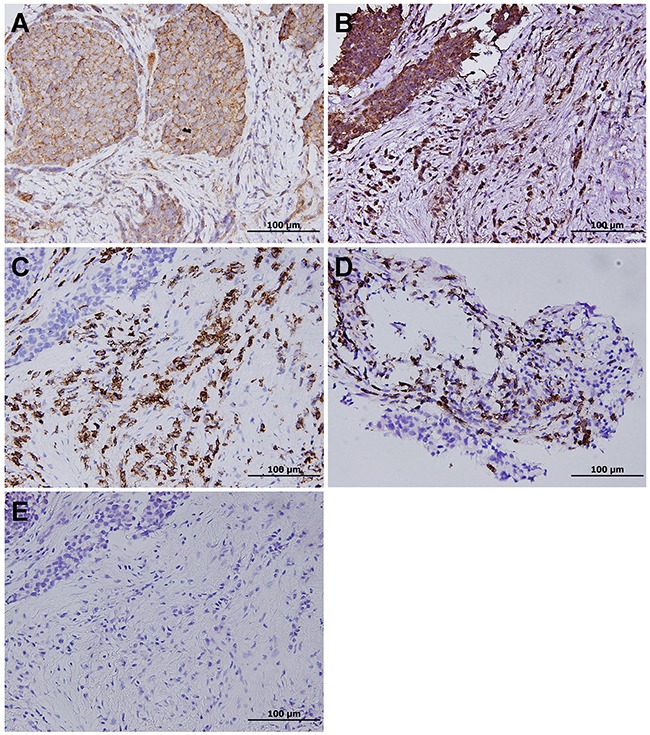
**A**. Positive PD-L1 immunohistochemical staining with a membranous pattern in small cell lung carcinoma. **B**. Besides, PD-L1 is expressed in tumor-associated lymphocytes, which stain positive for **C**. CD45 and **D**. the T-cell marker CD3, while negative for **E**. the macrophage marker CD68.

Immunostaining for PD-L1 was observed in the membrane of the tumor cells and stromal lymphocytes. Tumor PD-L1 membranous reactivity (Figure 2A) was detected in 145 of the 186 patients (78.0%). There were also PD-L1-positive monocytic cells (Figure 2B) in the adjacent stroma. These cells were immunoreactive to the common leukocyte IHC marker CD45 (Figure 2C) and the T-cell marker CD3 (Figure 2D), while negative for the macrophage marker CD68 (Figure 2E) and were considered tumor-associated lymphocytes (TILs). Varying numbers of TILs were noted and 101/186 cases (54.3%) showed PD-L1-positive TILs. The staining intensity was usually moderate to strong similar to what was observed in the tumor cells. The TILs were frequently located at the interface between carcinoma cells and the stroma, with occasional infiltration within the carcinoma cells.

### Correlations of PD-L1 expression with clinicopathologic features and survival

High PD-L1 expression was associated with advanced age (≥ 60 years) (P<0.001) and PD-L1 expression in TILs (P=0.001) (Table 1).

**Figure 3:**
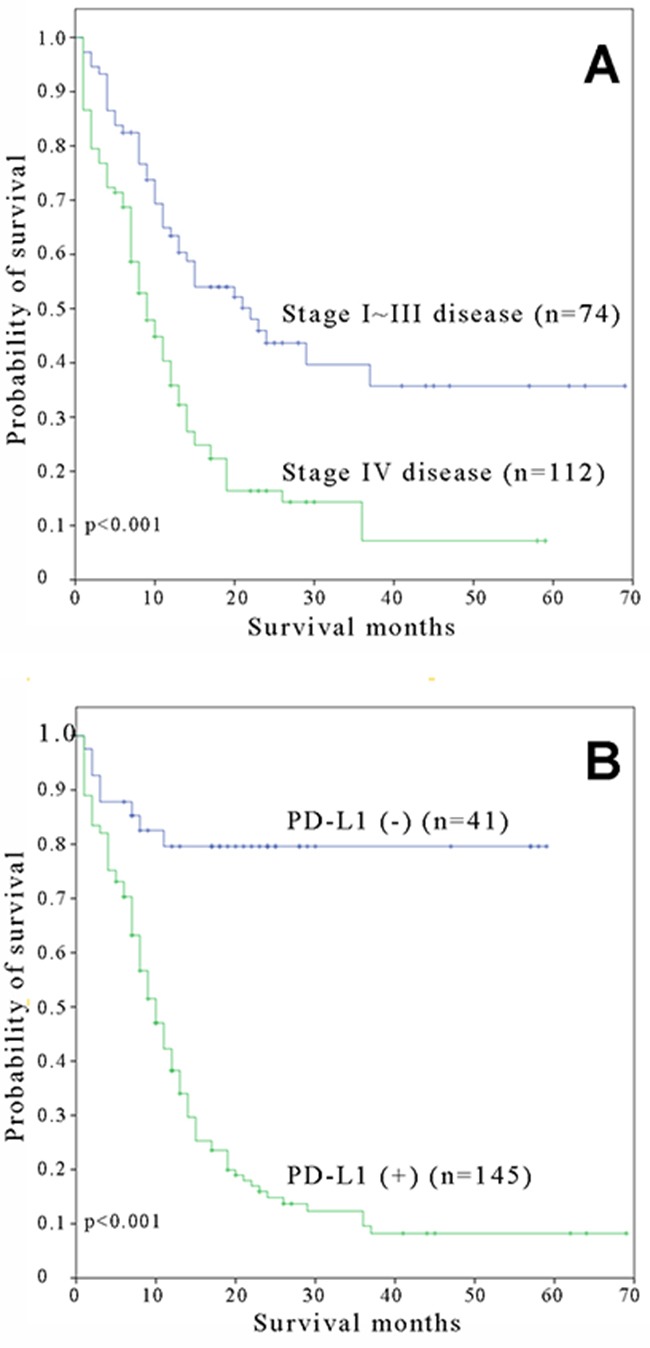
Kaplan-Meier overall survival curves for small cell lung carcinoma patients with A. stage I-III and stage IV and B. positive and negative expressions of PD-L1

Survival analysis using a Kaplan-Meier plots and log-rank tests identified three significant risk factors for poor overall survival: advanced age (P=0.001), stage IV diaease (P<0.001) and positive tumor PD-L1 expression (P<0.001) (Table 2). In the multivariate analysis, stage IV disease (Figure 3A) and positive tumor PD-L1 expression (Figure 3B) were significantly associated with poor overall survival (Table 3).

**Table 2:** Correlation between clinicopathologic factors and survival in patients with small cell lung cancer

Variables	Patient no.	No. of deaths (%) / 5-year OS (%)	*p* value
Patient number	186	125 (67.2)/19.8	
Age (year)			
<60	49	24 (49.0)/42.6	0.001
≥60	137	101 (73.7)/12.9	
Sex			
Male	167	114 (68.3)/18.8	0.392
Female	19	11 (57.9)/23.7	
Smoking status*			
Smoker	160	107 (66.9)/18.0	0.385
Nonsmoker	13	7 (53.8)/34.2	
Necrosis			
≥20%	53	36 (67.9)/16.8	0.636
<20%	133	89 (66.9)/21.0	
Stage			
Stage IV	112	86 (76.8)/7.2	<0.001
Stage I-III	74	39 (52.7)/35.7	
PNS			
Positive	101	68 (67.3)/13.8	0.791
Negative	85	57 (67.1)/24.2	
Serum LDH level			
Normal	88	60 (68.2)/23.0	0.476
Abnormal	98	65 (66.3)/17.0	
Serum CEA level			
Normal	130	89 (68.5)/20.2	0.647
Abnormal	56	36 (64.3)/19.6	
Tumor PD-L1 expression			
Positive	145	117 (80.7)/8.2	<0.001
Negative	41	8 (19.5)/79.6	
TIL PD-L1 expression			
Positive	101	65 (64.4)/17.5	0.977
Negative	85	60 (70.6)/22.5	

*No information in some of the patients

Abbreviations: PNS, paraneoplastic syndrome; PD-L1, programmed cell death ligand 1; TIL, tumor-infiltrating lymphocytes.

**Table 3:** Multivariate survival analysis of clinicopathologic features in patients with small cell lung cancer

Variables	Overall survival		
**HR**	**95% CI**	***p*** **value**
Stage			
Stage IV	1.000		
Stage I-III	0.476	0.322~0.703	<0.001
Tumor PD-L1 expression			
Positive	1.000		
Negative	0.168	0.081~0.345	<0.001

Abbreviations: PD-L1, programmed cell death-ligand 1

## DISCUSSION

SCLC is a heterogeneous and genetically complex disease with a very high mortality rate. The current standard of care includes concurrent chemoradiation with cisplatin and etoposide for stage I-III disease SCLC and a combination of platinum and etoposide or irinotecan for stage IV disease SCLC [13]. Despite the high chemosensitivity of these tumors, > 90% of patients with metastatic disease will experience disease relapse after a response and there are limited therapeutic options for these patients. Furthermore, despite therapeutic progress, the clinical benefit of new targeted therapies has been disappointing in SCLC [14]. The etiology and molecular events associated with this disease are mostly unknown. There are presently no available data indicating the optimal management of patients with SCLC.

For the past few decades, the choices for the systemic disease control of SCLC have been limited. Recently, many clinical trials have reported that PD-L1 blockade provides durable tumor control with minimal-related adverse events [7, 8]. The overexpression of PD-L1, as defined by IHC, in tumor cell membranes is one of the predictive biomarkers noted by previous studies [6, 9, 16–22]. Patients with positive PD-L1 expression in tumor cells have experienced improved clinical outcomes following anti-PD-L1-directed therapy [15, 23]. The recent phase III clinical trials of nivolumab documented that tumor PD-L1 expression could predict treatment response in non-squamous NSCLC [24] but not squamous cell carcinoma [25].

Contradictory to the results reported by Schulthesis et al., 145 of 186 cases (78.0%) of SCLC in this series showed positive membranous PD-L1 staining in tumor cells. Our results of frequent PD-L1 expression in SCLC are supported by two recent studies by Ishii et al [20]. and Komiya T, et al [22].

The reasons for the discrepancies among studies are unclear but may include the following: sample collection, methodology for tissue fixation, different antibodies, cut-off points and scoring methods. The cut-off value for PD-L1 positivity is a concern when interpreting the study results. IHC testing has yet to be standardized and optimal IHC assays have not been validated. The IHC antibody we used was also utilized in several studies on various neoplasms, including lymphoma, [27] lung cancer, [6, 16, 19, 28, 29] and head and neck cancer [17]. The cut-off value of 5% seemed to be a reasonable threshold for PD-L1 expression with our IHC antibody.

Our study revealed that tumor PD-L1 expression and a stage IV disease were significantly associated with poorer prognosis independent of the other factors examined. This result corresponds with previous studies showing that PD-L1 is associated with poorer prognosis in patients with NSCLC, [6, 30] esophageal carcinoma, [31] gastric carcinoma, [32] pancreatic carcinoma, [33] hepatocellular carcinoma, [34] renal cell carcinoma, [11] and ovarian carcinoma [35]. In contrast to our study, several previous studies reported that PD-L1 expression was associated with better prognosis in patients with NSCLC, [16, 28] SCLC, [20] breast cancer, [36] and malignant melanoma [37]. This discrepancy between the present study and these previous studies may be due to a number of reasons. First, the baseline characteristics of the lung cancer cases included in these studies are heterogeneous and the differences in PD-L1 expression in tumors with dissimilar histology or pathological stage may affect the outcome analysis. Second, the threshold for PD-L1 expression positivity has not been clearly defined, and reproducibility has not been formally assessed. Third, the techniques and detailed protocol may differ among these studies. For future clinical applications, further efforts to standardize a protocol for ascertaining PD-L1 expression are warranted.

PD-L1 staining on immune cells in lung cancer has been reported in a few studies [9, 15, 20, 21] with the prevalence ranging from 26-53%. In the present study, we found that 54.3% of cases showed PD-L1 expression in TILs. Recent studies demonstrated that the best clinical tumor responses to anti-PD-L1 therapy were associated with PD-L1 expression in tumor-infiltrating immune cells [15, 26]. Taken together, frequent PD-L1 expression in either tumor or immune cells in SCLC indicates that targeting the PD-1 axis holds a promise for the clinical treatment of SCLC.

SCLC has long been associated with PNS. Endocrine paraneoplastic disorders are characterized by ectopic production of peptide hormones and the neurological complications are related to antibody-mediated damage to the central nervous system. Hyponatremia is the most common PNS (up to 15%) [14] but the incidence was much higher (54.3%) in our series. It might be sensible to screen for SCLC-associated autoantibodies in possible clinical trials to reduce the risk of adverse events.

In conclusion, this large cohort study demonstrates that tumor PD-L1 expression in a membranous pattern is a common feature in SCLC, supporting the hypothesis that PD-L1 expression might lead to enhanced immune evasion by the tumor. High tumor PD-L1 expression was significantly correlated with TIL PD-L1 expression and stage IV disease. In addition, patients with positive PD-L1 expression had a poor clinical outcome. The results also indicate that clinical trials targeting PD-1 and/or PD-L1 may benefit patients with this otherwise difficult to treat disease, and the utility of such targeted therapeutics in clinical practice needs to be determined in randomized trials.

## MATERIALS AND METHODS

### Patient populations

The investigations were performed in a cohort of 186 individuals with SCLC, who were treated at the National Taiwan University Hospital between 1^st^ January 2010 and 31^st^ December 2015. The Hospital's Research Ethics Committee approved the study, and all the patients provided written informed consent. SCLCs was diagnosed according to the criteria set by the World Health Organisation [10]. Morphologic features were evaluated using hematoxylin and eosin-stained sections, and standard immunohistochemical (IHC) markers, such as neuroendocrine markers (chromogranin A, synaptophysin, and CD56), cytokeratins (pancytokeratin AE1/AE3 or CK7), Ki-67 and TTF-1 were analyzed.

Clinical data were tabulated from each patient's medical records and analyzed in conjunction with microscopic findings. The tumor samples used for IHC analysis included surgically resected specimens, bronchoscopy tissue and sonography or computed tomography (CT)-guided lung biopsy tissue from inoperable patients.

### Immunohistochemical analysis

For IHC staining of PD-L1 expression in tumor tissue, 4-μm-thick sections from each formalin-fixed, paraffin-embedded tissue blocks were de-waxed with xylene and rehydrated in a graded series of ethanol. For PD-L1 (a rabbit PD-L1 polyclonal antibody which has been used in several previous studies) [6, 16, 17, 19, 27, 28, 29] Proteintech group Inc., Chicago, IL, USA. The sections were incubated with the PD-L1 antibody (1:250 dilution) for 1h, and antigen retrieval was performed by autoclaving for 9 min at 121°C in citrate buffer, pH 6.0. The UltraVision Quanto Detection System HRP DAB (Thermo Fisher Scientific, Fremont, CA, USA) was used according to the manufacturer's instructions. The sections were counterstained with hematoxylin and then mounted.

PD-L1 immunostaining results were classified into two groups according to the intensity and extent of staining: (1) negative, no staining or staining detected in < 5% of the cells; and (2) positive, when membranous staining was present in ≥ 5% of the cells and the staining intensity was moderate to strong. The 5% threshold was based on a previous phase 1 trial of anti-PD-1 agents and studies of other malignances [8, 11, 12]. Two independent pathologists (C-T. W. and Y-L. C.) assessed PD-L1 expression.

### Statistical analysis

The correlation was correlated with various clinicopathologic parameters using Fisher's exact test. Overall survival (OS) was assessed using the Kaplan-Meier method, and the log-rank test was used for comparisons. Prognostic factors of OS were analyzed by univariate and multivariate analyses. Hazard ratios and 95% confidence intervals were calculated for all variables in the regression model. All tests were two sided, and P < 0.05 was considered significant. PASW Statistics 18.0 (IBM Corporation, Armonk, NY, USA) was used for all the analyses.
